# Manual Acupuncture and Laser Acupuncture for Autonomic Regulations in Rats: Observation on Heart Rate Variability and Gastric Motility

**DOI:** 10.1155/2013/276320

**Published:** 2013-11-21

**Authors:** Zhao-Kun Yang, Mei-Ling Wu, Juan-Juan Xin, Wei He, Yang-Shuai Su, Hong Shi, Xiao-Yu Wang, Ling Hu, Xiang-Hong Jing, Gerhard Litscher

**Affiliations:** ^1^Department of Meridians, Institute of Acupuncture and Moxibustion, Academy of Chinese Medical Sciences, 16 Nanxiaojie, Dongzhimennei, Beijing 100700, China; ^2^Stronach Research Unit for Complementary and Integrative Laser Medicine, Research Unit of Biomedical Engineering in Anesthesia and Intensive Care Medicine, TCM Research Center Graz, Medical University of Graz, Auenbruggerplatz 29, 8036 Graz, Austria

## Abstract

This study focused on the effects of laser acupuncture (LA) and manual acupuncture (MA) at different acupoints on gastric motility and heart rate variability (HRV) simultaneously to elucidate the site specific effects of acupoints and the correlation between changes of gastric motility and low frequency/high frequency (LF/HF) ratio. Gastric motility and HRV were recorded before and during MA or LA. Stimulating PC-6 or ST-36 significantly enhanced gastric motility, while BL-21 caused no changes. In contrast, MA or LA at CV-12 significantly suppressed gastric motility. Stimulating PC-6 or ST-36 significantly increased heart rate (HR), while CV-12 or BL-21 induced no significant changes of HR. Stimulating PC-6 significantly increased LF/HF, while ST-36, CV-12, or BL-21 induced no significant effects. These results indicated that there was acupoint specificity in the effects of acupuncture on gastric motility and HRV. The stimulatory effect of MA and LA at PC-6 and ST-36 on HR was associated with sympathetic activity. The stimulatory effect of MA or LA at PC-6 or ST-36 on gastric motility was associated with vagal activity. Laser needle can be used as an alternative stimulation therapy.

## 1. Introduction

Acupuncture has been used for regulating autonomic dysfunction in gastrointestinal and cardiovascular diseases. Manual acupuncture (MA) is a traditional method used widely in the world. With the development of technology, different kinds of acupuncture methods have been used in clinic. Laser acupuncture (LA) is a new technology stimulation with low energy and no pain, and it can also be used noninvasively instead of acupuncture needles; thus it becomes easily accepted by more and more patients especially in Europe and America [1].

MA or LA has been used for autonomic regulation [2]. Heart rate variability (HRV) has been used to evaluate the balance between the parasympathetic and sympathetic activity. Low frequency (LF) represents sympathetic activity, while high frequency (HF) represents parasympathetic or vagal activity. Therefore, LF/HF can be an indicator of the sympathovagal balance. In a previous study, different frequencies of violet LA on HRV were investigated. The result showed that there was a significant difference in mean HR and LF/HF ratio before, during, and after 2 or 100s violet LA [3]. It was also demonstrated that HR changed and HRV increased significantly during interstitial LA stimulation of PC-6 [4], but whether the effect of LA is the same as that of MA still remains unclear. In the present study, the effect of MA or LA at different acupoints such as PC-6 (Neiguan), ST-36 (Zusanli), CV-12 (Zhongwan), and BL-21 (Weishu), respectively, on HRV and gastric motility was investigated simultaneously in rats, to determine the specificity of acupoints related to the internal organs and if the effects were affected by the stimulating methods. 

## 2. Animals and Methods

### 2.1. Animal Preparation

Ten male healthy Sprague-Dawley rats (weight: 190–250 g, purchased from the Institute of Animals, China Academy of Chinese Medical Sciences) were kept in an animal house maintained at 24 ± 1°C, with a 12-hour light-dark cycle and free access to food and water for one day before the experiment. The animals were anesthetized with an intraperitoneal injection of 10% urethane (1.2 g/kg, Sigma-Aldrich, St. Louis, USA). Animals were sacrificed by an overdose of anesthetics after the study. The study was approved by the Institutional Animal Care and Use Committee of the China Academy of Chinese Medical Sciences and was in accordance with National Institutes of Health guidelines.

### 2.2. Gastric Motility Recording

After intraperitoneal injection of urethane, the animals were under deep anesthesia. A small longitudinal incision was made in the duodenum about 1 cm from the pylorus. A small balloon made of flexible condom rubber was inserted via incision of the duodenum into the pyloric area of rat and kept in position by tying the connecting catheter to the duodenum. Another catheter (inner diameter 1 mm) was also inserted into the same hole by incision in order to drain digestive juices secreted from the stomach. The balloon was filled with about 0.2-0.3 mL warm water to keep pressures at about 100 mmH_2_O. Pressure in the balloon was measured by a transducer through a thin polyethylene tube (1.5 mm in outer diameter) and then input into a polygraph amplifier (NeuroLog, NL900D; AutoMate Scientific, Berkeley, CA, USA). The signal was captured online and analyzed offline using a data acquisition system (Power-Lab/4s; AD Instruments, Colorado Springs, CO, USA) and Chart 5.2 software. Demifasting gastric motor activity was recorded as a control for at least 30 min before any stimulation. The gastric motility induced by stimulation was compared with the background activity in terms of integral (returns the integral of the selection, calculated as the sum of the data points multiplied by the sample interval). Rectal temperature was kept constant at around 37°C by a feedback-controlled heating blanket.

Gastric motility during stimulation was compared with background activity. If the change rates of gastric motility during or after stimulation were 15–20% of the basal activity, the response was considered to have an excitatory or inhibitory effect. The first stimulation was applied when gastric motility wave maintained stability, usually about one hour after the surgical procedure [5]. 

### 2.3. Electrocardiographic Monitoring

To collect electrocardiographic (ECG) data in rats, three needle electrodes were placed separately in subcutaneous muscles, in the left and right forelimbs and the left hindlimb separately [6]. The data for HRV analysis was derived from ECG recording using a special software (HRV module for Chart5, AD Instruments, Colorado Springs, CO, USA). The mean HR and LF/HF ratio of HRV were evaluated.

### 2.4. Stimulation

#### 2.4.1. Laser Acupuncture

LA was employed to stimulate PC-6, CV-12, ST-36, or BL-21 separately. PC-6 is located proximal to the accessory carpal pad of the forelimb, between the flexor carpi radialis and palmaris longus ligaments. CV-12 is located in the medioventral line, 3 mm above the umbilicus. ST-36 is located on the anterolateral side of the hindlimb near the anterior crest of the tibia below the knee under the tibialis anterior muscle. BL-21 is located at 5 mm lateral to the spinal process of the 12th thoracic vertebrae in rats [7]. The laser needle (length 35 mm, diameter 0.55 mm) was a Modulas needle (type: IN-Light, Schwa-Medico, Ehringshausen, Germany). It emits red laser light in continuous wave mode with a wavelength of 658 nm and an output power of 50 mW [4]. In this study, laser acupuncture was performed interstitially (needle penetration depth was 1–3 mm).

#### 2.4.2. Manual Acupuncture

MA was also performed at the four acupoints, respectively. For MA stimulation, sterile single-use needles (length: 15 mm, diameter: 0.3 mm; Huan Qiu, Suzhou, China) were inserted perpendicularly to the skin with a depth of approximately 3–5 mm at the acupoint. The needles were stimulated clockwise and counterclockwise at a frequency of 2 Hz. The stimulation was performed immediately after inserting the needle, continued for 3 minutes, and then withdrawn [8]. 

Any stimulation was only applied when the gastric motility and HRV had recovered to control state. The two signals before and during stimulations were recorded continuously, 180 s for each session. Two stimulation methods were performed randomly, with an interval of no less than 10 min.

### 2.5. Statistical Analysis

Quantification of gastric motility was studied by calculating the motility index (MI). The MI is equivalent to the area under the curve of motility recording. MI was calculated every 3 min before and during acupuncture using Power Lab software (AD instruments, Colorado Springs, CO, USA).

MI after EA or LA was compared with the background activity and expressed as % change of MI in each rat. The data obtained before and during treatment in the same group were compared by a paired *t*-test; different groups were compared by independent-sample *t*-test. *P* < 0.05 was considered as of a statistical significance. All data are expressed as mean ± standard error (SE).

## 3. Results

### 3.1. Gastric Motility under Resting Condition

The gastric motility of the rats was detected by recording the intragastric pressure. When the intrapyloric balloon pressure was increased to about 80–200 mmH_2_O, the rhythmic waves of contractions in the pyloric area were observed. With regard to gastric motor characteristics, both the changes of intragastric pressure and rhythmic contraction were noteworthy. Generally, the intragastric pressure represents the index of gastric tone motility and rhythmic contraction represents gastric peristalsis induced by circular muscle contractions, similar to slow wave of gastric motor activity. The pressure was maintained at about 100 mmH_2_O as baseline by expanding the volume of the balloon with warm water; rhythmic contractions occurred at a rate of four to six per minute.

### 3.2. Effects of MA and LA on Gastric Motility Induced by Different Acupoints

**Figure 1:**
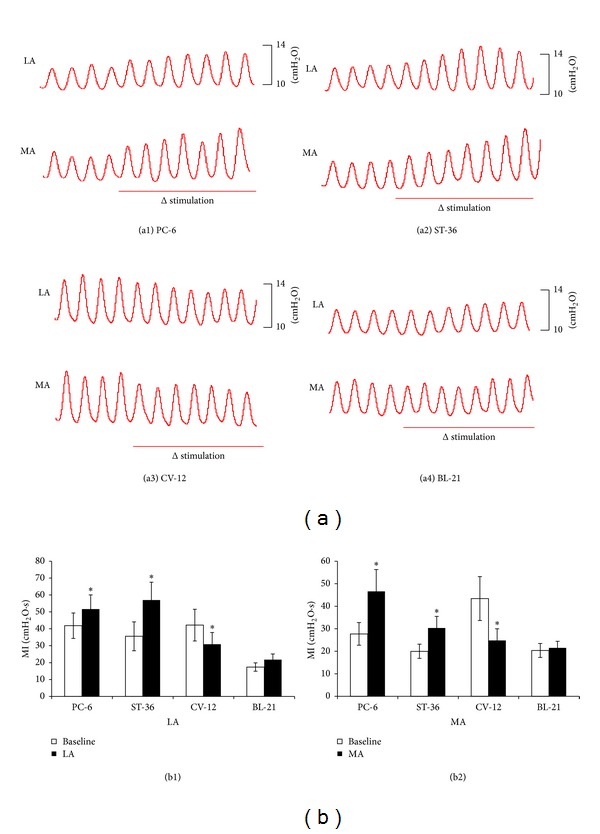
Effects of MA and LA on gastric motility induced by different acupoints. (a) Representative examples of the alteration of gastric contraction wave. (a1), (a2), (a3), and (a4) represent the stimulation of acupoints PC-6, CV-12, ST-36, and BL-21, respectively. (b1) Gastric motility index (MI) at baseline and during LA (*n* = 10 per group). (b2) MI at baseline and during MA (*n* = 10 per group). *P < 0.05, compared with baseline. Stimulation for 180 seconds is indicated by horizontal bars.

**Figure 2:**
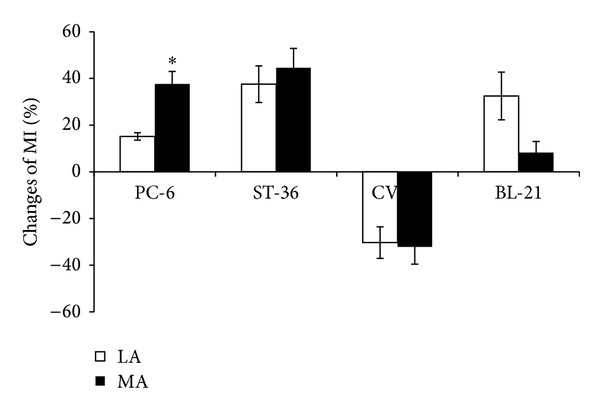
Comparison of changes of the MI induced by LA or MA at four acupoints, respectively (*n* = 10 per group). *P < 0.05, compared with LA.

The gastric motility was enhanced during LA or MA at PC-6.  Figure 1 shows that the MI increased from 41.86 ± 7.52 cmH_2_O to 51.67 ± 8.44 cmH_2_O· (*P* < 0.05) induced by LA; and it also increased from 27.72 ± 5.02 cmH_2_O· to 46.59 ± 9.73 cmH_2_O· by MA (*P* < 0.05). The gastric motility was also facilitated by LA or MA at ST-36 (LA: MI from 35.56 ± 8.56 cmH_2_O· to 56.98 ± 10.59 cmH_2_O· (*P* < 0.05) and MA: MI from 19.99 ± 3.17 cmH_2_O· to 30.32 ± 5.18 cmH_2_O· (*P* < 0.05)). In contrast, the gastric motility was inhibited by LA or MA at CV-12. The MI was inhibited from 42.19 ± 9.37 cmH_2_O· to 30.85 ± 6.99 cmH_2_O· by LA (*P* < 0.05), and for MA it decreased from 43.40 ± 9.74 cmH_2_O· to 24.81 ± 5.20 cmH_2_O· (*P* < 0.05). However, LA or MA at BL-21 did not induce significant changes in gastric motility (see Figure 1). The effect of MA at PC-6 on gastric motility was stronger than that of LA (LA: 15.12 ± 1.59% and MA: 37.62 ± 5.43%, *P* < 0.05) (Figure 2).

### 3.3. Effects of MA and LA at Different Acupoints on HRV

**Figure 3:**
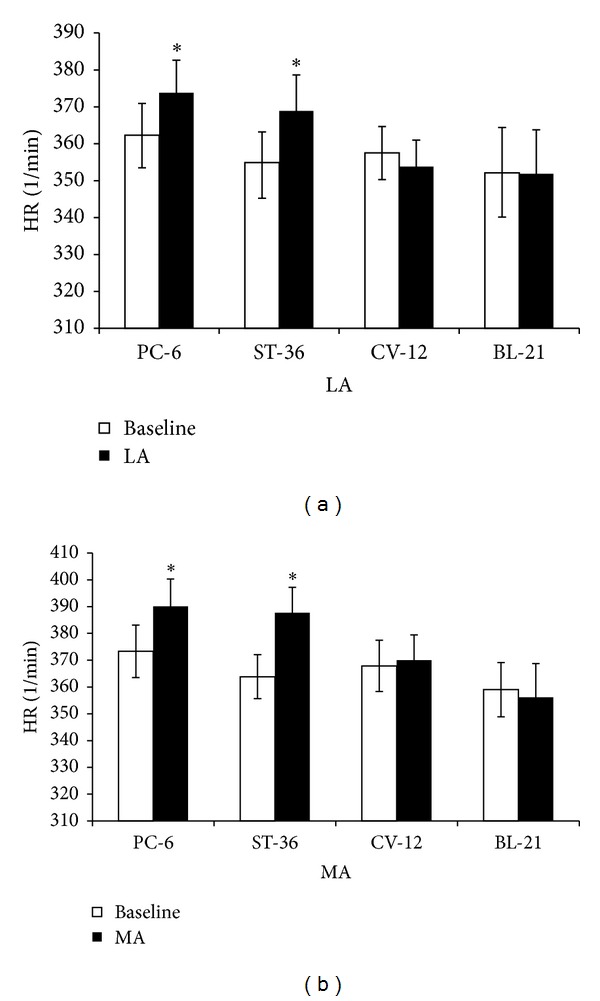
HR changes induced by LA or MA at four acupoints, respectively. (a) HR by LA at PC-6, ST-36, CV-12, or BL-21, respectively. (b) HR by MA at PC-6, ST-36, CV-12, or BL-21, respectively (*n* = 10 per group). *P < 0.05, compared with baseline.

**Figure 4:**
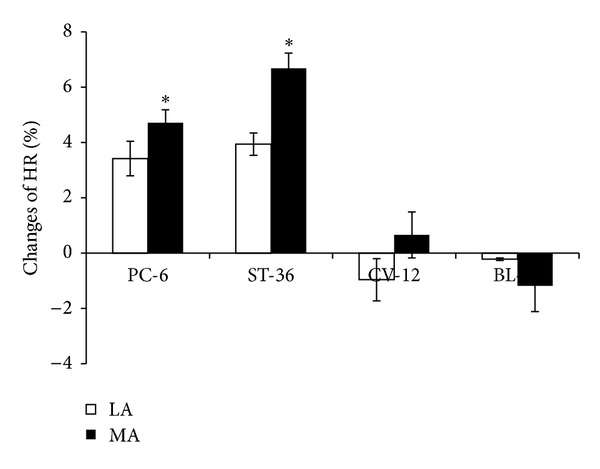
Changes of HR induced by LA or MA at four acupoints, respectively (*n* = 10 per group). *P < 0.05, compared with LA.

Both LA and MA stimulation at PC-6 induced an increasing effect on HR which was from 362.3 ± 8.6/min to 373.8 ± 8.8/min (LA, *P* < 0.05) and from 373.3 ± 9.8/min to 390.1 ± 10.2/min (MA, *P* < 0.05). LA or MA at ST-36 also increased HR from 355.0 ± 8.3/min to 368.9 ± 9.7/min (LA, *P* < 0.05) and from 363.9 ± 8.2/min to 387.7 ± 9.5/min (MA, *P* < 0.05). However, LA or MA at CV-12 or BL-21 produced no significant difference in HR (Figure 3). MA at PC-6 or ST-36 had a stronger influence on the change of HR than LA (*P* < 0.05) (Figure 4).

**Figure 5:**
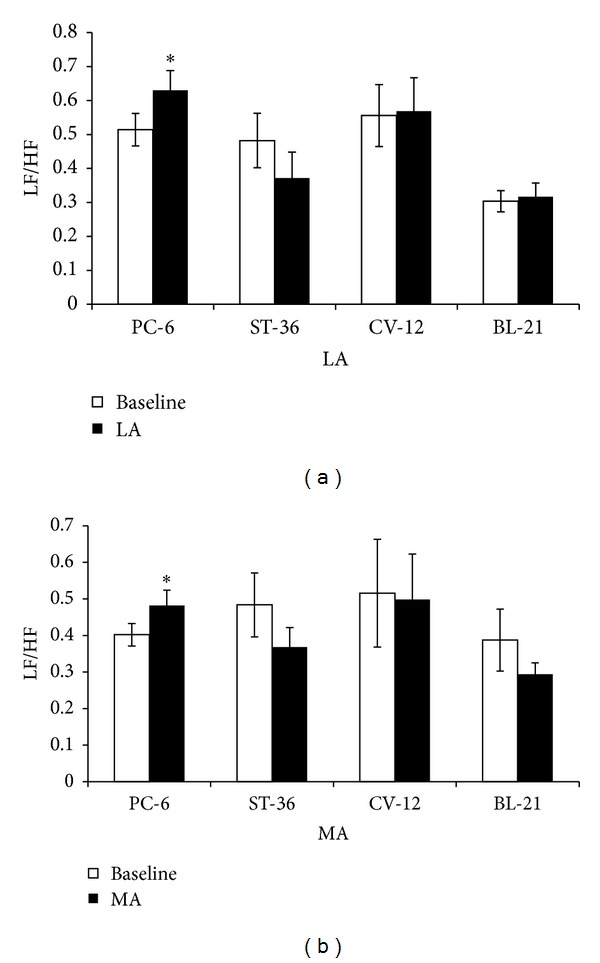
Change rate of LF/HF induced by LA or MA at four acupoints, respectively. (a) shows LF/HF by LA and (b) shows LF/HF by MA at PC-6, ST-36, CV-12, or BL-21, respectively (*n* = 10). *P < 0.05, compared with baseline.

**Figure 6:**
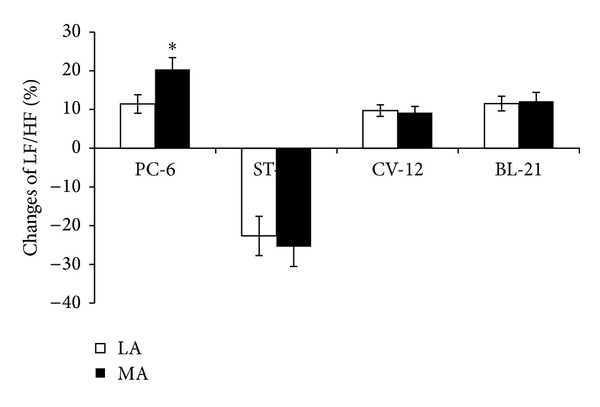
Changes of LF/HF induced by LA or MA at four acupoints, respectively (*n* = 10 per group). *P < 0.05, compared with LA.

The HF power provides an index of parasympathetic (vagal) activity, while LF/HF ratio is an indicator of sympathovagal balance. Both LA and MA stimulation at PC-6 induced an increase of LF/HF. The LF/HF ratio increased from 0.51 ± 0.048 to 0.63 ± 0.058 (LA, *P* < 0.05) and from 0.40 ± 0.03 to 0.48 ± 0.042 (MA, *P* < 0.05). However there was no significant difference in LF/HF induced by LA or MA at ST-36, CV-12 or BL-21, respectively, (Figure 5). The change rate of LF/HF induced by MA at PC-6 was stronger than that by LA (*P* < 0.05) (Figure 6).

## 4. Discussion

We previously showed that electroacupuncture (EA) at ST-36 caused gastric contractions, while EA at CV-12 caused gastric relaxations in rats, and there existed an “intensity-response” relationship between stimulation and effects on gastric motility. TRPV1 receptor was involved in the regulation process of EAS [5]. Previous studies also suggested that acupuncture at the lower limbs stimulated gastric motility via vagal cholinergic pathways, while acupuncture at the abdomen inhibited gastric motility via sympathetic pathways in rats [9]. Now more and more studies demonstrated that the effects of acupuncture at ST-36 and CV-12 on gastric motility were related to the autonomic nervous system [10].

The spectral analysis of HRV of ECG has been used to evaluate the balance between the parasympathetic and sympathetic activity in humans. Although many previous articles studied the effect of acupuncture on gastric motility [11] or HRV [12], only a few studies have assessed both parameters simultaneously especially in laser acupuncture. In the present study, the effects of MA or interstitial LA on gastric motility and HRV induced by stimulation of different acupoints were compared. The results showed that both MA and LA at PC-6 and ST-36 enhanced gastric motility and increased HR simultaneously, while at CV-12 suppressed gastric motility. LA or MA at PC-6 increased LF/HF, while MA or LA at the other stimulated acupoints had no significant difference on LF/HF. These results indicated that stimulations at different acupoints evoked different effects on gastric motor function and cardiac autonomic nerve function in anesthetized rats, which confirmed that there is acupoints specificity in regulating autonomic functions. Therefore, acupoint selecting is important in treating diseases.

Acupuncture as a somatic stimulation induced two kinds of reaction forms of alterations in visceral function including segmental and systemic regulation [13]. The segmental somatosympathetic reflex occurs between the surface and visceral organs of the same nerve segment. The central part of the spinal cord was involved in the sympathetic regulation. In the present study, either LA or MA at PC-6 increased HR which is perhaps mediated by the segmental somato-sympathetic reflex and enhanced gastric motility which is perhaps mediated by sympathetic vagal regulation. It has also been found that either LA or MA at ST-36 enhanced gastric motility which may be mediated by vagal regulation, whereas LA or MA at CV-12 suppressed gastric motility via segmental somatosympathetic reflex in the spinal level. 

Part of our results was not in accordance with previous studies [11]. Li and Wang showed that stimulation at CV-12 increased the vagal HRV component without affecting the dominant ECG frequency, while stimulation at BL-32 decreased the dominant ECG frequency without affecting the vagal HRV component [14]. It was also suggested that the stimulatory effect of electroacupuncture at ST-36 on gastric motility is associated with its stimulatory effect on vagal activity [15]. Further studies will focus on the question which acupoints are more important on autonomic function in terms of sympathetic and parasympathetic influence. 

As a modern technique, laser stimulation has been used in clinic and research of acupuncture. LA showed a clinically and statistically significant benefit with reducing symptoms of depression on objective measures including different clinical scores [16]. LA at ST-36 elicited significant antinociceptive effects against acetic-acid- and formalin-induced behavior in rats, and this effect is mediated by activation of the opioidergic and serotonergic (5-HT1 and 5-HT2A receptors) systems [17]. According to our results, both MA and interstitial LA induced similar effects on autonomic functions, while acupuncture at different acupoints induced different effects on autonomic functions, which indicates that acupoint position is more important than acupuncture stimulation method in treating diseases. Compared with MA, LA has a potential role in clinic because of its painlessness, noninvasiveness, and safety, making it an alternative method of MA for those who are afraid of manual acupuncture.

## 5. Conclusion

There is acupoint specificity in regulating autonomic functions. The stimulatory effect of MA and LA at PC-6 and ST-36 on HR was associated with sympathetic nerve activity. The stimulatory effect of MA and LA at PC-6 and ST-36 on gastric motility was associated with vagal activity. The choice of the acupoint is more important than the stimulation method in treating diseases by acupuncture. Laser needle can be used as a noninvasive alternative stimulation therapy.
